# New Method for Pure-Tone Audiometry Using Electrooculogram: A Proof-of-Concept Study

**DOI:** 10.3390/s18113651

**Published:** 2018-10-28

**Authors:** Do Yeon Kim, Jinuk Kwon, Joo-Young Kim, Ho-Seung Cha, Yong-Wook Kim, In Young Kim, Chang-Hwan Im

**Affiliations:** Department of Biomedical Engineering, Hanyang University, Seoul 04673, Korea; dkim9681@hanyang.ac.kr (D.Y.K.); kowm2000@naver.com (J.K.); jykim1026@bme.hanyang.ac.kr (J.-Y.K.); chayo89@bme.hanyang.ac.kr (H.-S.C.); kim3863215@naver.com (Y.-W.K.); iykim@hanyang.ac.kr (I.Y.K.)

**Keywords:** audiometry, electrooculogram (EOG), pure-tone audiometry, spatial sound movement, objective assessment

## Abstract

Precise and timely evaluation of an individual’s hearing loss plays an important role in determining appropriate treatment strategies, including medication and aural rehabilitation. However, currently available hearing assessment systems do not satisfy the need for an objective assessment tool with a simple and non-invasive procedure. In this paper, we propose a new method for pure-tone audiometry, which may potentially be used to assess an individual’s hearing ability objectively and quantitatively, without need for the user’s active response. The proposed method is based on the auditory oculogyric reflex, where the eyes involuntary rotate towards the source of a sound, in response to spatially moving pure-tone audio stimuli modulated at specific frequencies and intensities. We quantitatively analyzed horizontal electrooculograms (EOG) recorded with a pair of electrodes under two conditions—when pure-tone stimuli were (1) “inaudible” or (2) “audible” to a participant. Preliminary experimental results showed significantly increased EOG amplitude in the audible condition compared to the inaudible condition for all ten healthy participants. This demonstrates potential use of the proposed method as a new non-invasive hearing assessment tool.

## 1. Introduction

Hearing loss is one of the most common health problems affecting the lives of elderly adults [1,2,3,4] and is becoming more prevalent across all age groups, owing to higher exposure to excessive noise from the workplace or use of personal music players [5]. Therefore, diagnosing an individual’s hearing loss, particularly at an early stage, would play a critical role in providing appropriate treatment.

Audiometry measures frequency-specific hearing thresholds [6] and classifies an individual’s hearing ability based on type, severity, and configuration [5,7]. Depending on classification results, hearing care professionals including audiologists, hearing aid specialists, and otolaryngologists, suggest different treatment options such as medications, surgical treatments, or the use of hearing aids. For instance, with age-related hearing loss, different aural rehabilitation strategies are recommended for the best use of hearing aids, to facilitate communication and promote maximum quality of life [8,9,10]. Because hearing loss can hinder speech and language developments and subsequently cause cognitive, emotional, behavioral, and academic problems in young children [11,12,13], identification of hearing loss during childhood is also essential to supply appropriate training programs, such as lip reading and sign language. Therefore, diagnosis of hearing loss should not only be precise but also readily accessible to both adult and pediatric patients.

There are several audiometry systems used to evaluate hearing ability and many researchers have tried to develop accurate, reliable, and fast methods to estimate hearing thresholds. Among them, pure-tone audiometry (PTA) testing has been the most widely used for more than six decades and is considered the “gold standard” in this field [14,15,16]. PTA has several advantages over other systems. This test is fully non-invasive and requires only a simple and quick diagnostic procedure. Furthermore, development of computer-based or online app-based PTA programs has broadened its accessibility [17,18,19,20].

However, the current PTA system has some drawbacks that may introduce errors into hearing threshold levels. Since hearing threshold levels are estimated from the subject’s behavioral and psychological data, the traditional PTA system may be (1) dependent on certain variables, such as attention span and reaction time, (2) open to manipulation by examiner’s mistakes or malingerers, and (3) ill-suited for young children who are not mature enough to participate [21,22,23]. As mentioned previously, since hearing care professionals determine proper treatment strategies based on classification of hearing loss and audiogram patterns [24], unreliable assessment of hearing loss may lead to ineffective treatment results. To circumvent these potential disadvantages, it is of great necessity to develop objective tools that are less dependent on the subject’s active participation or co-operation while maintaining advantages of the conventional method.

In this paper, we propose a concept for a novel hearing assessment method based on quantitative analysis of an electrooculogram (EOG) [25], which has potential to be developed as a more objective audiometry method than the conventional observer-based psychoacoustic procedure. We tried to develop a hearing assessment method that does not require any voluntary responses from subjects, by which they do not need to determine whether they heard a sound (especially a small, vague sound). We generated spatially moving pure-tone sound stimuli with different intensities and frequencies and observed eye movements induced by the auditory oculogyric reflex (involuntary rotation of the eyeballs toward the source of a sound) using horizontal EOGs, recorded while a user is hearing each moving sound with his/her eyes closed. As an initial proof-of-concept study, we investigated the possibility of the proposed method by comparing EOG waveform patterns acquired when pure-tone stimuli were either inaudible or audible to each participant.

## 2. Materials and Methods

### 2.1. Participants

A total of ten healthy young participants (seven males and three females, 24.0 ± 2.31 years in age) participated in this study. All participants were students attending Hanyang University and had no medical history or clinical diagnosis of brain injury or tinnitus. Each participant was verbally informed of the detailed experimental protocol and signed a consent form prior to the experiment. Monetary reimbursement was provided to each participant after completion of the experiment. This study was approved by the Institutional Review Board of Hanyang University and was conducted in accordance with the Declaration of Helsinki. All experimental procedures and methods were carried out within approved guidelines.

### 2.2. Experimental Design

The experimental procedure was designed to investigate the relationship between eye movements when the participants listened to spatially moving pure-tone auditory stimuli and pure-tone thresholds (PTTs) obtained through conventional PTA tests.

The entire experiment was divided into two parts and conducted by two examiners. In the first part of the experiment, denoted as part I and was composed of eight repeated sessions, the proposed EOG-based audiometry method was tested. Each session started with a 5 s verbal introduction (in Korean, delivered using an automated voice), asking the participants to close their eyes and concentrate on the moving sound, followed by 16 consecutive EOG recording trials. The duration of each trial was set to 6 s and inter-trial interval was set to a random duration of 2–5 s. In the first trial (Trial 1), no sound was presented for the “no-sound condition”. Then, different combinations of frequencies (1000, 2000, and 4000 Hz) and sound intensities (30, 40, 50, 60, and 70 dBA) created 15 different sound stimuli. For Trails 2–6, 1000 Hz pure-tone sound stimuli with five different sound intensities were presented in a randomized order. For Trials 7–11, 2000 Hz stimuli were presented, and for Trials 12–16, 4000 Hz stimuli were used.

In the second part of the experiment (denoted as part II), two examiners assessed each participant’s air-conduction hearing threshold at the same frequencies used in part I (i.e., 1000, 2000, and 4000 Hz) using a conventional PTA with a modified Hughson-Westlake ascending-descending procedure through a “up 5 dB–down 10 dB” technique. All participants underwent the entire test procedure twice per examiner. Results were double-blind during the experiments and collected afterwards by one examiner. Note that all participants participated in both experiments I and II.

### 2.3. Spatially Moving Pure-Tone Stimuli

**Figure 1:**
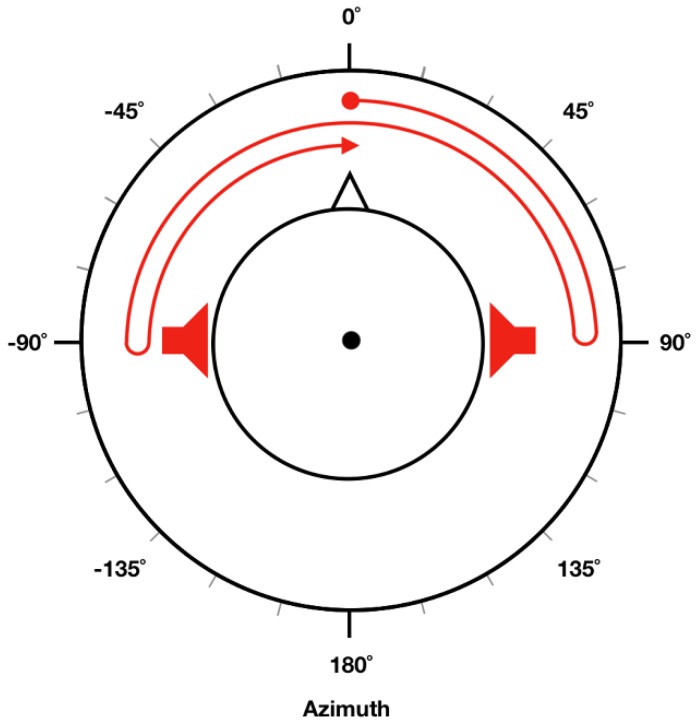
Generation of spatially moving pure-tone stimuli. In order to make a pure-tone sound stimulus move along the trajectory shown as a red-headed arrow, different sound intensities were applied to each ear based on current location of the sound source.

Spatially moving sound stimuli presented in the experiments were generated using MATLAB R2016b (MathWorks, Natick, MA, USA). We first generated pure-tone sound signals with corresponding frequency and intensity (referred to as “target intensity” in this study) for each trial. In Figure 1, trajectory of the moving sound is illustrated as a red arrow, where the sound source started from a position straight in front of the head (0° azimuth) and then moved toward the right side (right ear, 90° azimuth). It then turned back and moved toward the left side (left ear, −90° azimuth). Finally, it returned to the original position and repeated this circle. To make participants feel that the sound source was actually moving, different intensities of pure-tone sound stimuli were given to the left and right ear [26] depending on the current location of the sound source. When the sound source was located in front of the head, a pure tone 10 dB lower than target intensity was presented to both ears. When it was located in the right ear (90° azimuth), a pure tone at target intensity was presented only to the right ear (no sound presented in the left ear). When it was located in the left ear (−90° azimuth), a pure tone at target intensity was presented only to the left ear (no sound was presented in the right ear). To create a continuously moving pure-tone stimuli, we evenly divided a 90° segment (either 0° to 90° or −90° to 0°) into six sub-segments by placing five points between either 0° and 90°, or −90° and 0°. Different sound intensity (in dBA) levels were given to each ear based on the current (virtual) location of the sound source, which could be readily evaluated using a linear interpolation of sound intensities at 0°, 90°, and −90°. In our experiment, each circle (0° → 90° → −90° → 0°) lasted for 3 s; namely, stimuli moved at a frequency of 1/3 Hz. Therefore, a cycle was repeated twice during each 6-s EOG recording trial.

### 2.4. Apparatus

**Figure 2:**
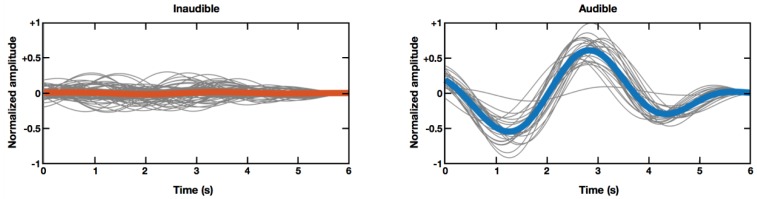
An example of grand averaged (bold) horizontal EOG and single trial EOGs (grey) recorded under different conditions: inaudible condition (**left**) and audible condition (**right**). These signals were recorded from a single participant (participant seven).

To record a horizontal EOG signal, a pair of flat Ag/AgCl electrodes were placed approximately 2.5 cm away from the lateral canthi of the eyes (see Figure 2a in Reference [25]). The EOG was recorded using a multi-channel biosignal recording system (BioSemi ActiveTwo, Amsterdam, The Netherlands) at a sampling rate of 2048 Hz. Each electrode was referenced and grounded by a common mode sense (CMS) electrode and a driven right leg (DRL) electrode attached at the left and right mastoids respectively. A StimTracker (Cedrus Corporation, San Pedro, CA, USA) system was used to synchronize the recording system and computer generating the sound stimuli.

The Digital Audiometer program (Clinical Professional Version 6.0a; Digital Recordings, Halifax, NS, Canada), a computer-based PTA, was used for part II. Before part II started, the program was calibrated to 60 dB at 1000 Hz using a sound-level meter (Cesva SC-30; CESVA Instruments SLU, Barcelona, Spain).

Each participant was required to wear noise reduction earplugs to raise their individual hearing threshold level above background noise (20–25 dBA) and to obtain a sufficient amount of data for inaudible conditions. Both parts of the experiment were conducted in a soundproof booth with the participants’ eyes closed. Examiners managed experiments outside the soundproof booth and monitored each participant through a window. All instructions and pure-tone stimuli were given through headphones (K271 MkII, AKG Acoustics, Vienna, Austria).

### 2.5. EOG Signal Processing

EOG data obtained from the experiments was processed using MATLAB R2016b. EOG data were down-sampled to 512 Hz and a horizontal EOG shift was calculated by subtracting electric potential recorded at the right EOG electrode from that at the left EOG electrode. The mean value (DC offset voltage) of each EOG epoch (trial) was then subtracted from the EOG shift to remove the gross DC offset component. A fourth-order Butterworth zero-phase band-pass filter with cut-off frequencies of 0.2 and 0.4 Hz was applied to extract eye movement components, owing to the moving sound source from the pre-processed EOG. Note again that the speed of the moving sound stimulus was set at 1/3 Hz.

### 2.6. Data Analysis

To compare EOG waveform patterns in inaudible and audible sound intensities, we first classified EOG data into two groups based on PTT results from part II. Note that we only considered trials with intensities below or above PTA thresholds. In other words, trials with exactly the same intensity as the PTA threshold were discarded from analysis.

Differences of EOG waveforms recorded under two conditions (inaudible and audible), were quantitatively compared using EOG amplitude, defined as the maximum absolute value in each trial. Sounds with intensities above and below PTTs were referred to as audible and inaudible sounds respectively. EOG amplitudes were normalized by dividing them by the EOG amplitude acquired for 70 dB and the 2000 Hz condition. Note that all participants could hear the auditory stimulus with 70 dB intensity and 2000 Hz frequency. In each session, normalized EOG amplitudes were averaged for inaudible and audible conditions, respectively. Since the total number of repeated sessions was eight, eight EOG amplitude values obtained for the inaudible condition and eight EOG amplitude values for the audible condition were statistically compared using Wilcoxon signed rank test. SPSS ver. 24.0 (Microsoft; Armonk, NY, USA) was used for statistical analyses.

## 3. Results

Figure 2 shows grand averaged horizontal EOG waveforms (thick colored lines), as well as single-trial EOG waveforms (thin gray lines) under two different conditions (inaudible sound condition versus audible sound condition), recorded from participant seven. As shown in Figure 2, normalized EOG amplitudes recorded when inaudible sound sources were moving, were much smaller than those recorded when audible sound sources were moving. This example demonstrates that EOG amplitude might be a promising feature to determine whether a subject was actually hearing the moving sound stimuli.

**Figure 3:**
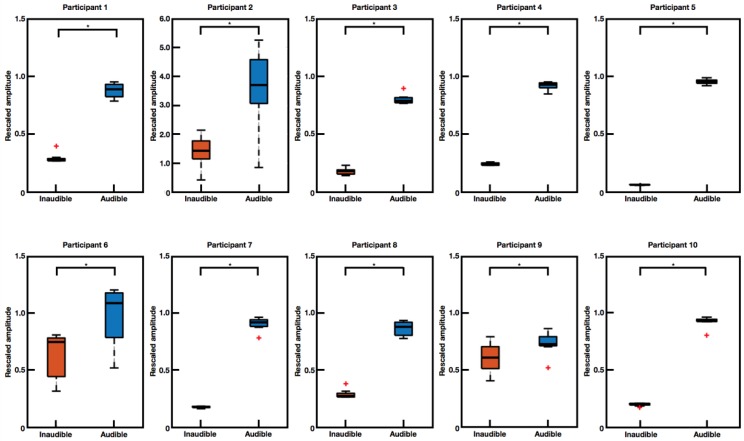
Box plots of rescaled (normalized) amplitudes for inaudible and audible conditions. Wilcoxon signed rank test showed a statistically significant difference between two conditions—inaudible and audible conditions—in all subjects (Bonferroni corrected *p* < 0.05, marked as *). Note that box plots for participant two have different scaling ranges.

Figure 3 shows the comparison of normalized EOG amplitudes for inaudible and audible conditions, which were evaluated for each participant. For statistical analysis we applied the Wilcoxon signed rank test because all sample distributions did not pass the one-sample Kolmogorov-Smirnov test of normality (*p* < 0.05). After applying the Bonferroni multiple comparison correction, statistical analysis exhibited significant difference (*p* < 0.05) between inaudible and audible conditions in all participants. These results suggest that the proposed EOG-based audiometry method has potential to be used as a new tool for assessing hearing loss.

## 4. Discussion

The aim of this study was to introduce a new audiometry system that uses a biosignal EOG, indirectly evoked by an individual’s attention to a rotating sound. We found that an individual’s eyeballs involuntarily rotate when he or she is effortlessly listening to a spatially moving pure-tone sound stimuli with different intensities and frequencies with his or her eyes closed. This phenomenon is a kind of auditory oculogyric reflex (involuntary rotation of the eyes toward the source of a sound) and can be readily measured using two horizontal EOG electrodes attached outside the lateral canthi of the eyes. Results of quantitative analysis demonstrated that EOG amplitude was significantly increased when a pure-tone sound was presented at audible frequencies and intensities, compared to EOG amplitude when the sound stimulus was not audible.

Although the present study showed the possibility of using EOG to assess an individual’s hearing loss, several issues remain to be addressed in future studies. First, the current system needs to be developed further to classify an individual’s audibility with high accuracy. Establishing standardized cut-off values or parameters to discriminate between audible and inaudible conditions seems to be ideal. However, large inter-individual variability of EOG potential level [27,28] needs to be overcome. Indeed, results in Figure 3 showed overlapping EOG amplitude distributions in three of ten participants. Use of additional features (e.g., use of phase information) and adoption of advanced classification algorithms might be potential solutions for this issue. For instance, EOG waveforms look synchronized when an audible sound source is moving, whereas they become out of phase when the participant cannot hear the sound, as shown in Figure 2.

Second, normalized EOG amplitude could be dependent on the reference value (maximum EOG amplitude for a pure tone stimulus with 2000 Hz frequency and 70 dB intensity). For instance, amplitude box plots of participant two and six showed relatively longer whiskers and tails in Figure 3 than those for other participants. Since we normalized EOG amplitudes by division with the reference value of each session, a wrong reference value (due to mistake of the subjects, e.g., opening the eyes) could introduce wrong normalized values throughout the entire trial of the same session. For example, a relatively larger scale of EOG amplitude in participant two originated from reference errors. Repeated measurements of the reference trial may reduce such errors.

Third, the current method cannot assess the hearing ability of each ear (either left or right ear) separately. To implement such a system, it may be necessary to adjust sound intensity for each ear to different levels when generating moving sound stimuli. However, this procedure would require more test trials, eventually leading to increased test time. Development of more efficient testing schemes would be necessary to make the proposed method more practical. Indeed, we recorded EOG signals for all possible combinations of frequencies and intensities, rather than following the most common PTA procedure, namely, a modified Hughson-Westlake ascending-descending method. Reducing the duration of each trial is also required to enhance overall efficiency of the proposed method. Although only three frequencies corresponding to speech understanding were tested in this study, testing a wider range of frequencies, including those used in standard PTA would be necessary in future investigations. Most importantly, general applicability of the proposed EOG-based audiometry needs to be verified by testing with hearing impaired patients, such as elderly people who have general degenerative processes of the neurologic system and children.

Despite these limitations, the present proof-of-concept study demonstrated the potential of a new PTA system based on EOG, as a new non-invasive hearing assessment. After improving overall performance of the proposed audiometry system, it would be necessary to compare advantages and disadvantages between the proposed method and conventional objective audiometry methods, such as brainstem evoked response audiometry (BERA) and the otoacoustic emission (OAE) test. BERA is a hearing assessment method that uses small auditory evoked potentials recorded from a few electrodes attached to the scalp surface. The brainstem auditory evoked potential (BAEP) method is composed of seven waves and is clinically analyzed based on morphological characteristics such as absolute latency, wave amplitude, and inter-peak interval latency. Although both BERA and the proposed audiometry have a common advantage over conventional PTA in that they do not require the subject’s active participation, the proposed EOG-based audiometry is expected to be implemented more easily and more cheaply than BERA, considering that amplitude of EOG is generally much larger than that of BAEP. Because of the small amplitude of BAEP, repeated presentations of auditory stimuli (more than several hundred) are required to acquire clean BAEP waveforms [29]. OAE is a sound caused by motion of sensory hair cells in the cochlea, either spontaneously or due to external auditory stimulation. An OAE test is widely used as an objective indicator of healthy cochlear function especially for new-born hearing tests as it is simple and efficient. As the OAE test specializes in impairment of cochlea function, it is frequently used in conjunction with PTA testing [30]. Therefore, it would be interesting future to apply both the OAE test and proposed audiometry method simultaneously to assess hearing loss of a subject.

Please note that the proposed hearing assessment method did not fully overcome all problems of conventional PTA. For example, the proposed method can also be dependent on attention of the subjects. In addition, EOG signals might be contaminated by movement-related artefacts when the proposed method is used for hearing assessments of young children, who might have difficulty staying still during the recording session. Therefore, further work should be done to implement a more reliable and efficient EOG-based audiometry system that can be used in practical scenarios. In addition, development of a low-cost portable EOG recording device incorporated with an audio headset would foster better accessibility of the proposed audiometry system.

## References

[1] Kim J.S. (2015). Prevalence and factors associated with hearing loss and hearing aid use in Korean elders. Iran J. Public Health.

[2] Deepthi R., Kasthuri A. (2012). Validation of the use of self-reported hearing loss and the Hearing Handicap Inventory for elderly among rural Indian elderly population. Arch. Gerontol. Geriatr..

[3] Lin F.R., Thorpe R., Gordon-Salant S., Ferrucci L. (2011). Hearing loss prevalence and risk factors among older adults in the United States. J. Gerontol. A Biol. Sci. Med. Sci..

[4] Mathers C.D., Stein C., Fat D.M., Rao C., Inoue M., Tomijima N., Bernard C., Lopez A.D., Murray C.J.L. (2002). Global Burden of Disease 2000: Version 2 Methods and Results.

[5] Zahnert T. (2011). The differential diagnosis of hearing loss. Dtsch. Arztebl. Int..

[6] Fukuda D.K., Ramsey M.J. (2013). Audiometry. Encyclopedia of Otolaryngology, Head and Neck Surgery.

[7] Dobie R.A., Van Hemel S., National Research Council (US) Committee on Disability Determination for Individuals with Hearing Impariments (2004). Hearing Loss: Determining Eligibility for Social Security Benefits.

[8] Dalton D.S., Cruickshanks K.J., Klein B.E.K., Klein R., Wiley T.L., Nondahl D.M. (2003). The impact of hearing loss on quality of life in older adults. Gerontologist.

[9] Li-Korotky H.-S. (2012). Age-related hearing loss: Quality of care for quality of life. Gerontologist.

[10] Samy R.N., Honaker J.A. (2013). Hearing Loss Rehabilitation for Acoustic Neuroma Patients.

[11] Cho Lieu J.E. (2004). Speech-language and educational consequences of unilateral hearing loss in children. Arch. Otolaryngol. Head. Neck Surg..

[12] Shojaei E., Jafari Z., Gholami M. (2016). Effect of early intervention on language development in hearing-impaired children. Iran. J. Otorhinolaryngol..

[13] Stelmachowicz P.G., Pittman A.L., Hoover B.M., Lewis D.E., Moeller M.P. (2004). The importance of high-frequency audibility in the speech and language development of children with hearing loss. Arch. Otolaryngol. Head. Neck Surg..

[14] Barlow C., Davison L., Ashmore M., Services S.A. (2015). Variation in tone presentation by pure tone audiometers: The potential for error in screening. EuroNoise.

[15] Maclennan-Smith F., Swanepoel D.W., Hall J.W. (2013). Validity of diagnostic pure-tone audiometry without a sound-treated environment in older adults. Int. J. Audiol..

[16] Roeser R.J., Buckley K.A., Stickney G.S., Roeser R.J., Valente M., Hosford-Dunn H. (2000). Pure Tone Tests.

[17] Foulad A., Bui P., Djalilian H. (2013). Automated audiometry using apple iOS-based application technology. Otolaryngol. Head. Neck Surg..

[18] Swanepoel D.W., Mngemane S., Molemong S., Mkwanazi H., Tutshini S. (2010). Hearing assessment-reliability, accuracy, and efficiency of automated audiometry. Telemed. J. E-Health..

[19] Corry M., Sanders M., Searchfield G.D. (2017). The accuracy and reliability of an app-based audiometer using consumer headphones: Pure tone audiometry in a normal hearing group. Int. J. Audiol..

[20] Vinay, Svensson U.P., Kvaløy O., Berg T. (2015). A comparison of test-retest variability and time efficiency of auditory thresholds measured with pure tone audiometry and new early warning test. Appl. Acoust..

[21] Muller G. (1987). Audiometry in young children. Can. Fam. Physician.

[22] Wooles N., Mulheran M., Bray P., Brewster M., Banerjee A.R. (2015). Comparison of distortion product otoacoustic emissions and pure tone audiometry in occupational screening for auditory deficit due to noise exposure. J. Laryngol. Otol..

[23] Sabo M.P., Winston R., Macias J.D. (2000). Comparison of pure tone and transient otoacoustic emissions screening in a grade school population. Am. J. Otol..

[24] Franks J.R., Goelzer B., Hansen C., Sehrndt G. (2001). Hearing Measurement.

[25] Lee K.-R., Chang W.-D., Kim S., Im C.-H. (2017). Real-Time “Eye-Writing” Recognition Using Electrooculogram. IEEE Trans. Neural Syst. Rehabil. Eng..

[26] Grothe B., Pecka M., McAlpine D. (2010). Mechanisms of sound localization in mammals. Physiol. Rev..

[27] Singh H., Singh J. (2012). Human Eye Tracking and Related Issues: A Review. Int. J. Sci. Res. Publ..

[28] Davis J.R., Shackel B. (1960). Changes in the Electro-Oculogram Potential Level. Br. J. Ophthalmol..

[29] Esteves M.C.B.N.E., Dell’ Aringa A.H.B., Arruda G.V., Dell’ Aringa A.R., Nardi J.C. (2009). Brainstem evoked response audiometry in normal hearing subjects. Braz. J. Otohinolaryngol..

[30] Kemp D.T. (2002). Otoacoustic emissions, their origin in cochlear function, and use. Br. Med. Bull..

